# Functional modulation of RAGE activation by multimeric S100B using single-domain antibodies

**DOI:** 10.1016/j.jbc.2024.107983

**Published:** 2024-11-13

**Authors:** Margarida C. Simões, Joana S. Cristóvão, Els Pardon, Jan Steyaert, Günter Fritz, Cláudio M. Gomes

**Affiliations:** 1 BioISI – Instituto de Biosistemas e Ciências Integrativas, Faculdade de Ciências, Universidade de Lisboa, Lisboa, Portugal; 2 Departamento de Química e Bioquímica, Faculdade de Ciências, Universidade de Lisboa, Lisboa, Portugal; 3 Structural Biology Brussels, Vrije Universiteit Brussel (VUB), Brussels, Belgium; 4 VIB-VUB Center for Structural Biology, VIB, Brussels, Belgium; 5 Department of Cellular Microbiology, University of Hohenheim, Stuttgart, Germany

**Keywords:** protein structure and folding, antibody fragments, receptor for advanced glycation end, S100 proteins, protein biophysics and interactions

## Abstract

S100B is a multifunctional protein primarily found in the brain, where it plays crucial roles in cell proliferation, differentiation, and survival. It has intracellular and extracellular functions and, depending on S100B levels, can exhibit both neurotrophic and neurotoxic activities, both mediated by the receptor for advanced glycation end products (RAGEs). Here, we report the discovery and characterization of nanobodies (Nbs) targeting dimeric and tetrameric S100B, which are the two most abundant oligomeric functional forms of the protein, aiming to modulate S100B-mediated RAGE activation. Two Nbs were selected for detailed structural and functional studies and found to bind tetrameric S100B with high affinity, as determined by biolayer interferometry (BLI) analysis and size-exclusion chromatography–stable binary complex formation. Structural and docking analyses revealed preferential contact sites of Nbs with S100B regions implicated in interactions with RAGE, namely residues at the interfacial cleft on dimeric S100B and at hydrophobic cleft formed by the association of two homodimeric units in the tetramer. In accordance, assays in SH-SY5Y cells showed that Nbs modulate the RAGE-mediated neurotrophic activity of S100B by hindering its functional interactions with the receptor. BLI competition assays between tetrameric S100B and the RAGE-VC1 domain confirmed that Nbs selectively block S100B-mediated RAGE engagement, in agreement with cell activation experiments. These findings highlight Nbs as powerful tools for elucidating molecular and cellular mechanisms through the modulation of S100B and RAGE functions, inspiring potential therapeutic applications.

S100B belongs to the Ca^2+^ binding S100 proteins family and is one of the most abundant soluble proteins in the brain. S100B is a multifunctional protein with intracellular and extracellular functions, which are related to the regulation of cellular processes, including protein phosphorylation, cell growth and motility, and cell survival (1, 2). At physiological conditions, S100B is neuroprotective acting as a prosurvival protein, triggering neurite outgrowth and cell proliferation and exerting chaperone functions (3, 4). Upon exacerbated expression, the protein is secreted and exhibits alarmin-like functions that can culminate in cell apoptosis (1). The neurotrophic and neurotoxic effects of S100B are mediated by engagement with the receptor for advanced glycation end products (RAGEs) (3). RAGE is a key signaling molecule in the innate immune system and is involved in the onset and sustainment of inflammation (5). It is a transmembrane protein formed by three extracellular immunoglobulin-like domains (V, C1, and C2), a transmembrane helix, and a short signal domain located at the C-terminal end (6).

S100B (10.7 kDa) occurs primarily as a homodimer but is also found in the brain in functional higher-order multimeric states (tetramers, hexamers, and octamers) with enhanced biological activities (7, 8, 9). Indeed, tetrameric S100B binds with higher affinity to RAGE than the dimeric and tetrameric S100B promotes RAGE-mediated increase in cell viability in HeLa (7) and SH-SY5Y (10) cells. Also, tetramerization of S100B and Cu-mediated polymerization resulted in improved anti-aggregation activity toward Aβ42 aggregation (8, 9). From a structural viewpoint, Ca^2+^ induces a substantial conformational change of the S100B homodimer with an ample rotation of helix IV and exposure of a hydrophobic surface and interfacial cleft formed between monomers. On the other hand, tetrameric S100B derives from the association of two homodimeric units resulting in an extended hydrophobic surface even in the apo state. These structural features mediate the functions of S100 proteins in general and of S100B in particular, highlighting the considerable functional versatility of these proteins based on their conformational plasticity and oligomerization properties (2, 11, 12).

Given its involvement in biological processes related to neurological and neurodegenerative diseases, inflammatory conditions, cellular proliferation, and metastasis, there is considerable interest in discovering modulators of S100B function. In fact, small-molecule inhibitors were already developed to interfere with S100B interactions with target proteins (13, 14, 15, 16). Pentamidine-related inhibitors demonstrated to inhibit the interaction between p53 and S100B, restoring p53 transcriptional activity in cancer cells (17, 18). Pentamidine also showed to decrease neuroinflammation and prevent neuronal loss in an Alzheimer's disease mouse model, suggesting that these inhibitors can also prevent S100B and RAGE interaction (15). However, most of these compounds have inherent cytotoxicity and biodelivery issues. Unlike small-molecule inhibitors, protein biologics, such as single-domain antibodies (or nanobodies [Nbs]), emerge as a potent class of therapeutics (19, 20). Nbs are the smallest antigen-binding domain of Camelid heavy-chain antibodies and have many beneficial properties for their application as research tools and biologics because of their small size (≈14 kDa), the possibility for recombinant production, genetic manipulation, and high affinity and specificity to their targets, allowing them to bind to structural and difficult-to-access epitopes (19). Therefore, with this study, we sought to develop Nbs raised against oligomeric S100B to generate molecular tools to regulate key biological processes and disease conditions in which S100B is implicated. Toward this goal, we here describe the generation and characterization of a library of anti-S100B Nbs that bind with low nanomolar affinity to both dimeric and tetrameric S100B and block the RAGE-mediated neurotrophic activity of S100B by competing with RAGE for S100B binding at the RAGE-VC1 domain.

## Results and discussion

### Generation of anti-S100B Nbs

**Figure 1:**
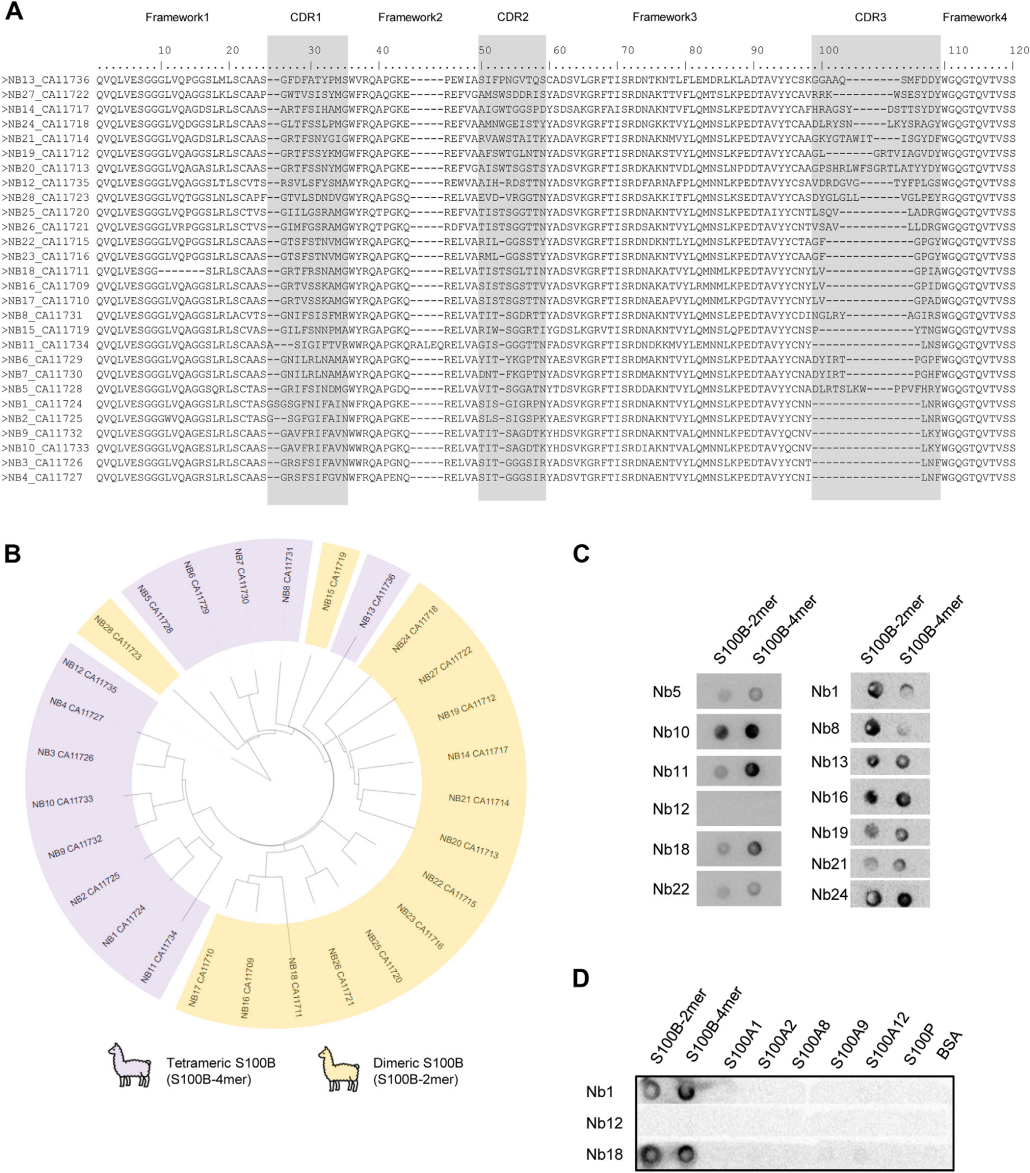
**Library of anti-S100B nanobodies (Nbs).***A*, multiple sequence alignment of the amino acid sequences of 28 anti-S100B Nbs. The three complementary-determining regions (CDR1, 2, and 3) of the anti-S100B Nbs were assigned according to the AbM definition (37, 38). *B*, tree analysis (*circle tree*) and Nb grouping according to immunization against tetrameric (*purple*) and dimeric (*yellow*) S100B. *C*, dot-blot analysis of the reactivity of a panel of anti-S100B Nbs against dimeric (S100B-2mer) and tetrameric (S100B-4mer) S100B. *D*, dot-blot analysis of reactivity of Nb1, 12, and 18 against S100s proteins (S100B dimer, S100B tetramer, S100A1, S100A2, S100A8, S100A9, S100A12, and S100P) and bovine serum albumin (BSA) as a negative binding control. See the Experimental procedures section for details.

Under physiological conditions, S100B primarily occurs as a homodimer that can self-assemble into functional tetramers, hexamers, and octamers. Tetrameric S100B is the most prevalent oligomer, which, unlike the higher-order oligomers, is formed irrespective of Ca^2+^ binding, is stable, and does not dissociate into dimers (7). To generate a library of Nbs against these species, as Nbs raised *in vivo* recognize discontinuous amino acid segments of the native protein conformation, llamas were immunized with both pure recombinant human dimeric and tetrameric S100B, following standard protocols (21). Phage libraries displaying the anti-S100B Nbs were generated from the RNA extracted from peripheral blood lymphocytes collected from two different llamas immunized separately with the S100B dimer (S100B-2mer) or S100B tetramer (S100B-4mer). Nbs were enriched through phage display *via* three rounds of panning against the immobilized antigen. Individual colonies were isolated, and *Escherichia coli* periplasmic extracts containing antigen-specific Nbs were screened for antigen binders using ELISA. Sequencing of the VHH genes from the positive signals resulted in a library of 28 anti-S100B Nbs, with 15 obtained from immunization with dimeric S100B and 13 from immunization with tetrameric S100B (Fig. 1*A*). Comparison of the selected Nb sequences reveals some clustering according to the immunizing species although with pannings done in parallel the possibility of crossed identifications cannot be ruled out; nevertheless, the fact that some branches comprise sequences obtained from the two series of immunization suggests that the raised Nbs may be able to recognize epitopes in both conformers (Fig. 1*B*).

### Expression and selectivity of anti-S100B Nbs

The anti-S100B Nbs were then expressed in *E*. *coli* and purified from periplasmic extracts using metal affinity chromatography. Except for Nb28, all VHH constructs were expressed and purified at reasonable yields, of at least 10 mg/L culture. A subset of 13 Nbs was then selected to confirm the recognition of native S100B by using dot-blot analysis (Fig. 1*C*). Although the Nbs were raised separately upon inoculation with dimeric and tetrameric S100B, all Nbs equally recognized both quaternary structures of S100B, except for Nb12, which recognized neither, suggesting a substantially weaker binding affinity. This indicates that the Nbs generated against the S100B-2mer and S100B-4mer recognize identical epitopes present in both conformers. Indeed, tetrameric S100B results from the relatively symmetrical association of a pair of S100B dimers (7). From the VHH library, we then selected for detailed studies Nb1 and Nb18, which are expressed at high yields (29 and 18 mg/L of culture) and were generated, respectively, from immunization with the S100B tetramer and dimer. Nb12 was also included in the panel as a weaker binder and potential negative control. Given the high structural homology between S100B and other members of the S100 protein family, and considering that Nbs recognize epitopes, we used dot-blot analysis to confirm that Nb1, Nb12, and Nb18 selectively recognizes S100B and not any of the other tested S100 proteins (S100A1, S100A2, S100A8, S100A9, S100A12, or S100P) nor bovine serum albumin (BSA), added as negative control (Fig. 1*D*).

### Nb structure, folding, and stability

**Figure 2:**
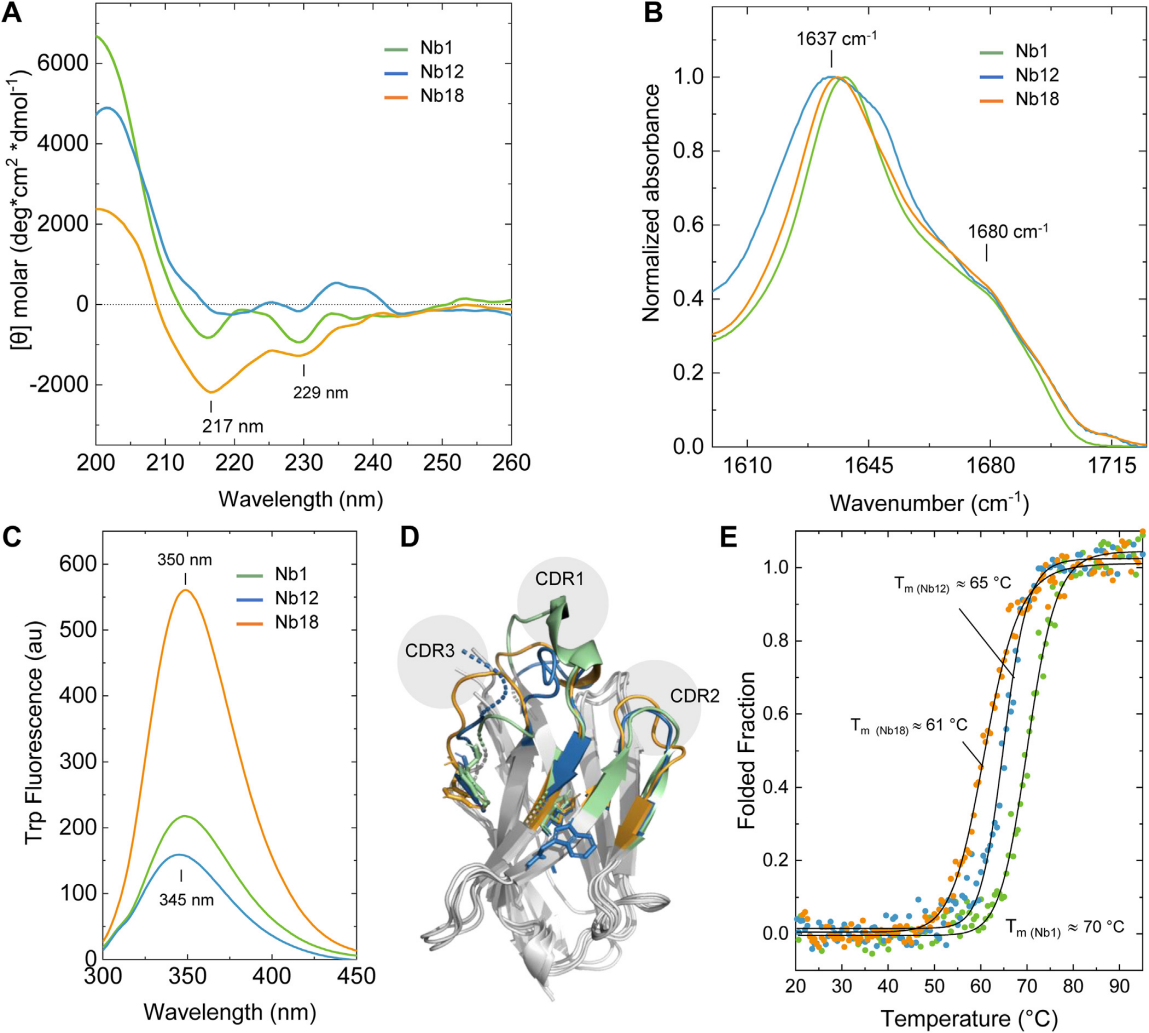
**Structural and biophysical analysis of three anti-S100B nanobodies (Nbs).***A*, far-UV CD, (*B*) ATR-FTIR, and (*C*) Trp emission spectroscopic analysis of Nb1, 12, and 18. *D*, structural models of the Nbs illustrating the common fold overlaid in *gray* and the Trp residues and complementary-determining regions color coded: Nb1 (*green*), Nb12 (*blue*), and Nb18 (*orange*). *E*, Nb thermal denaturation curves monitored by Trp fluorescence spectroscopy and estimated melting temperatures (Tm). See the Experimental procedures section for details.

We next characterized the structure, folding, and stability properties of the three selected Nbs. Nb structure consists of nine β-strands organized in a four-stranded β-sheet and a five-stranded β-sheet, which are connected by three loops containing the three complementary-determining regions (CDRs) (22). As expected for the β-sheet-rich immunoglobulin fold, the far-UV CD revealed weak negative bands centered in the 217 to 230 nm region and intense positive bands peaking toward 200 nm (Fig. 2*A*).

Complementary analysis by ATR-FTIR evidenced amide I bands centered in the 1620 to 1640 region, maxima around 1637 cm^-1^, and shoulders at 1680 cm^-1^ compatible with antiparallel β-sheets and β-turns (Fig. 2*B*). Trp fluorescence spectroscopy revealed emission spectra with maxima centered in the 345 to 350 nm region, in agreement with relatively solvent-exposed aromatic moieties (Fig. 2*C*). Although Nb1 and N18 have both two Trp residues in structurally conserved positions, Nb18 has higher emission; Nb12 contains a third Trp in the framework 2 region (Fig. 2*D*). Thermal stability analysis assessed by tryptophan fluorescence revealed melting temperatures within the typical expected range for Nbs (22), with Nb1 (T_m_ ≈ 70 °C) being slightly more stable than Nb12 (T_m_ ≈ 65 °C) and Nb18 (Tm ≈ 61 °C) (Fig. 2*E*); similar results were obtained with CD spectroscopy (Fig. S1). This observation is consistent with the fact that Nb1 has the shortest CDR3 among the three Nbs, which is compatible with the correlation of shorter CDR3 loops and increased stability because of decreased structural flexibility, although there is not a clear consensus on this matter (23).

### Affinity and structural analysis of Nb–S100B complexes

**Figure 3:**
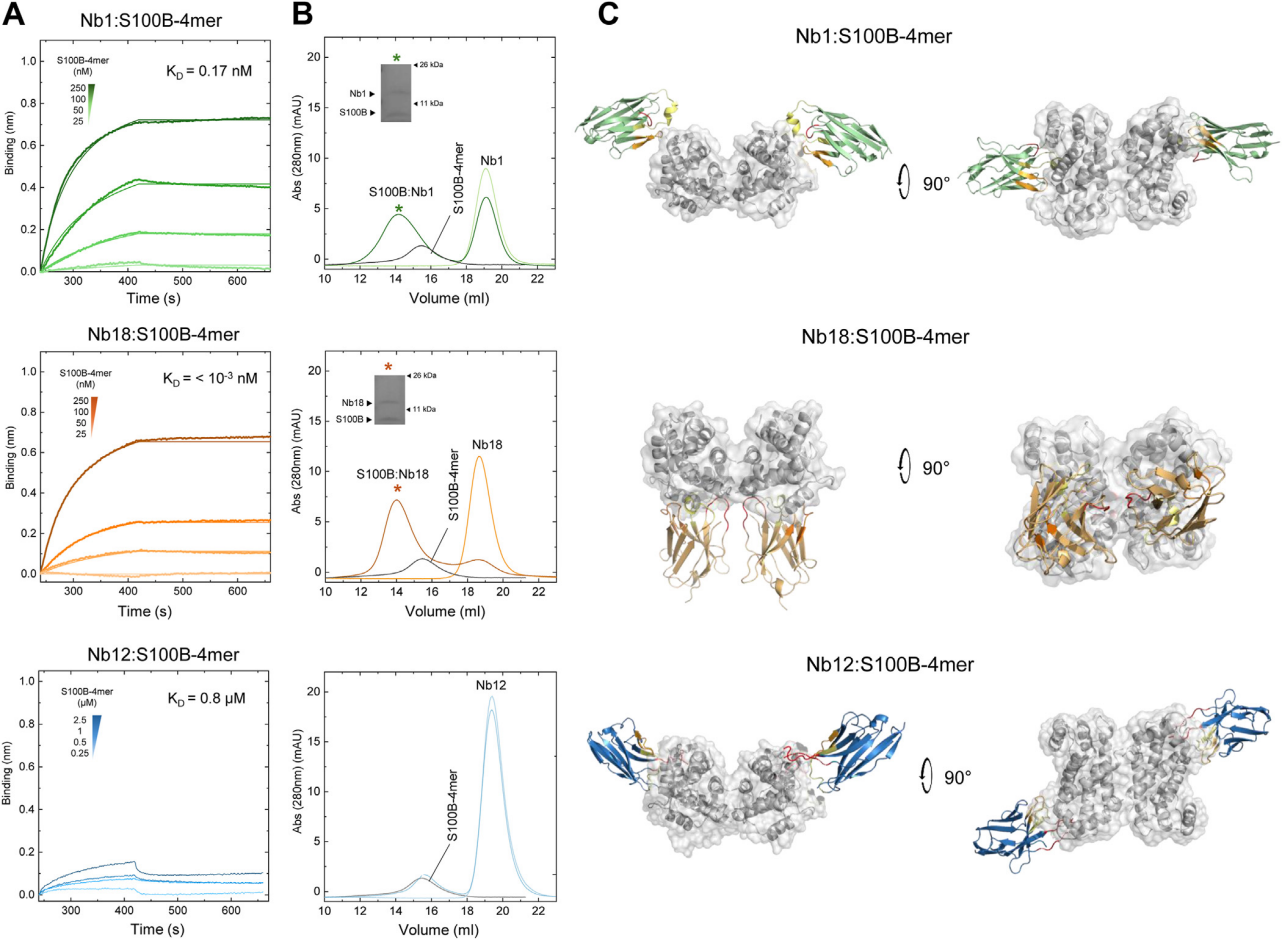
**Binding and structural analysis of three anti-S100B nanobodies (Nbs) to S100B-4mer.***A*, BLI sensograms and fitting of Nb binding to S100B-4mer. *B*, chromatograms obtained from size-exclusion chromatography resolved Nbs, S100B-4mer and binary complexes (for Nb1 and Nb18); *inset* depicts lane from an SDS-PAGE obtained from the peak corresponding to the complex (∗) to identify the presence of Nbs and S100B. *C*, docking models of complexes between Nbs (*colored*) and S100B-4mer (*gray*); because of the symmetry of the S100B-4mer, binding to two equivalent regions are highlighted. See Experimental procedures section for details. BLI, biolayer interferometry.

Next, we performed BLI measurements to determine the binding affinity of the selected anti-S100B Nbs to S100B. The results obtained show that Nb1 and Nb18 bind with high affinity to both tetrameric S100B (Fig. 3*A*) as well as to dimeric S100B (Fig. S2*A*), albeit at higher affinity for the former. In fact, no dissociation is observed upon binding of Nb18 to S100B-4mer, compatible with a *K*_*D*_ value below 10^-3^ nM. These estimates were obtained by fitting the data to a 1:1 binding model, which showed an excellent fit, indicating that the binding stoichiometry for the Nb complexes is indeed 1. Consistent with the dot-blot analysis, Nb12 exhibited a much weaker binding affinity to both S100B oligomers, binding to S100B dimer with a K_D_ ≈ 4 μM and to the tetramer with a K_D_ ≈ 0.8 μM.

To verify if high-affinity binding between Nbs and S100B oligomers would result in the formation of stable Nb–S100B complexes, we performed size-exclusion chromatography (SEC). For this, we incubated dimeric and tetrameric S100B with the different Nbs and checked whether this resulted in the formation of stable Nb–S100B complexes. Indeed, Nb1 and Nb18 formed stable binary complexes and eluted earlier in SEC at higher molecular mass. The presence of Nbs and S100B was confirmed by SDS-PAGE analysis of the peak fraction (Fig. 3*B* and S2*C*). We also assessed if Nb1 and Nb18 binding could be influenced by Ca^2+^ binding to dimeric and tetrameric S100B, as Ca^2+^ binding to S100 proteins induces conformational changes that expose hydrophobic regions known to mediate protein–protein interactions (17). A similar analysis using Ca^2+^ bound S100B in both quaternary states showed that Ca^2+^ binding to S100B does not substantially affect the formation of stable complexes with any of the tested Nbs (Fig. S3). This suggests that the Nbs are recognizing epitopes in S100B that are not significantly distinct between apo- and Ca^2+^ bound states. As expected, Nb12 did not form stable complexes with neither dimeric nor tetrameric S100B (Fig. 3*B*).

We then sought to gain structural insights into the possible binding sites for the Nbs in tetrameric S100B, which is a relevant functional oligomer for instance in what concerns RAGE engagement (7). For this, we used ClusPro 2.0 to perform unbiased *in silico* docking studies between Nb1, Nb12, and Nb18 and Ca^2+^ bound tetrameric S100B obtained from the X-ray structure of human Ca^2+^ loaded S100B octamer (7). Nb structures were modeled on NanoBodyBuilder2 (24). The best docking solutions obtained indicate that the three Nbs have distinct modes of interaction (Fig. 3*C* and Fig. S4). Nb1 interactions mainly involve residues from the CDR1 and CDR2 regions that are predicted to associate *via* hydrophobic and electrostatic interactions with residues forming the interfacial cleft of the dimeric unit formed by helices H_III_ and H_IV_. On the other hand, Nb18 engages all CDR residues in interactions modeled to occur preferentially in the central extended hydrophobic cleft formed by H_III_ and H_IV_ from the two homodimeric units composing the tetramer. Interestingly, the regions of S100B predicted to be involved in binding to Nb1 and Nb18, which mainly include the hinge and C-terminal regions, are known to play crucial roles in interactions with target proteins. These regions are also less conserved across the S100 protein family (11, 17, 25), which supports our observation of absence of cross-reactivity in the dot-blot experiments. Nb12-mapped interactions involved all CDR residues and the C-terminal regions of helices H_I_, H_III_, and H_IV_ and connecting loops.

### Nbs modulate RAGE-mediated S100B neurotrophic activity

**Figure 4:**
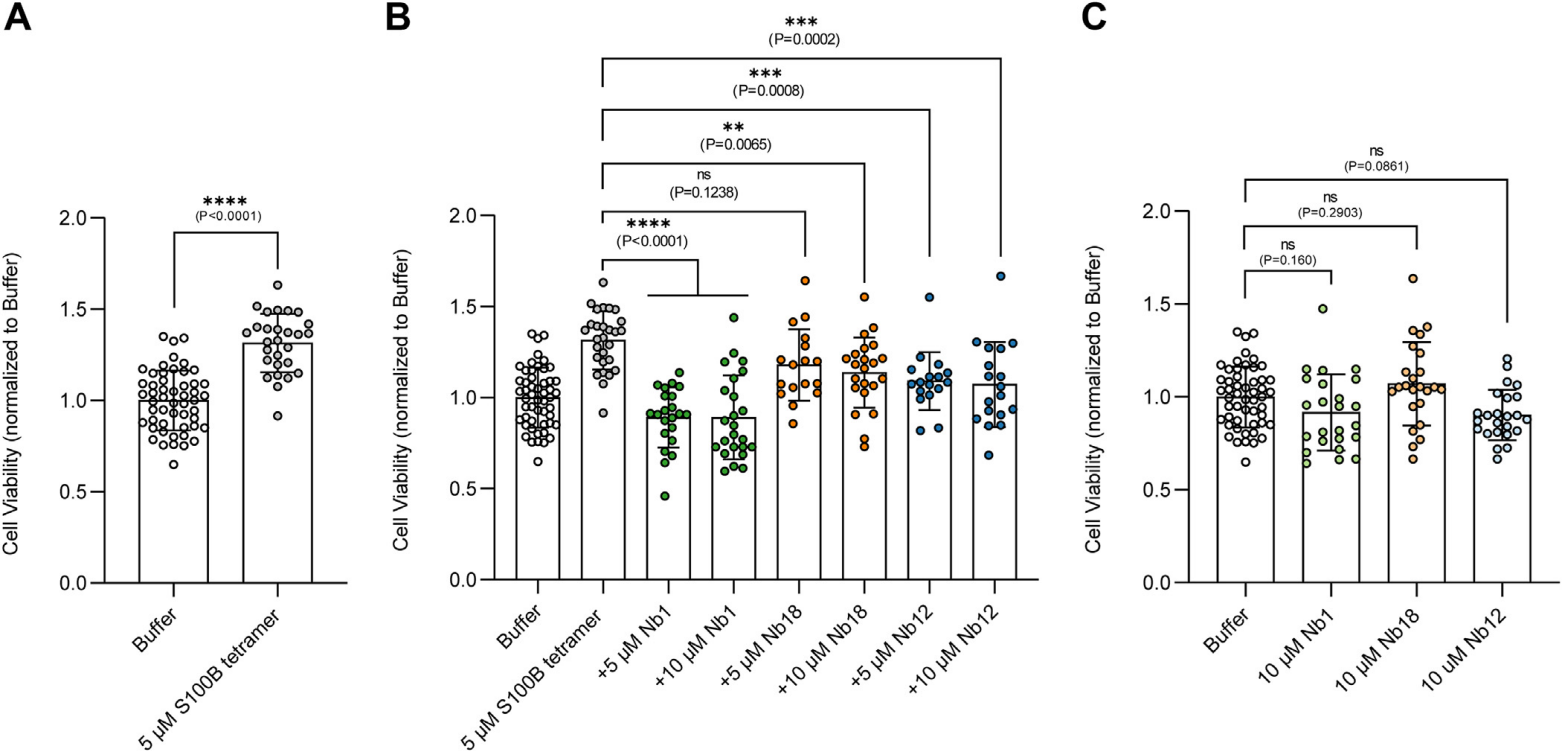
**Nanobodies (Nbs) modulate S100B neurotrophic activity in SH-SY5Y cells.***A*, cell viability assay (normalized to the control vehicle—buffer) of cells treated with (*A*) S100B tetramer (5 μM). *B*, S100B tetramer (5 μM) in the presence of Nb1 (5 or 10 μM), Nb18 (5 or 10 μM), or Nb12 (5 or 10 μM). *C*, Nb1, Nb12, or Nb18 (10 μM). All experiments were performed at least three times with at least six replicates each, and statistical significance was calculated through one-way ANOVA after normality test; *p-*values are indicated in the figure.

Extracellular S100B interacts with RAGE in various cell types, producing different outcomes—either beneficial or detrimental, depending on its concentration, the type of cell, and the microenvironment. As a first approach to test the hypothesis that Nbs modulate RAGE activation by S100B, we tested their effects on S100B's neurotrophic activity in SH-SY5Y cells, which is mediated by RAGE (10). Previous reports showed that treatment of SH-SY5Y cells with micromolar S100B increases cell viability in a RAGE-dependent manner (10). To test this effect, we treated serum-starved SH-SY5Y cells with tetrameric S100B. Consistent with previous observations, we observed a significant increase in cell viability mediated by S100B (Fig. 4*A*). Next, we compared the effect of tetrameric S100B in combination with Nb1, Nb12, and Nb18 (Fig. 4*B*). The treatment of cells with a combination of tetrameric S100B and Nb1 restored cell viability to control levels, suggesting a strong inhibitory effect of this Nb on the S100B–RAGE interaction. A milder reversion of the cellular effects was observed with Nb18. Nb12 also restored the increase in cell viability, which was surprising given its low binding affinity (K_D_ = 0.8 μM) to tetrameric S100B.

This result suggests that the weak interaction of Nb12 with S100B might occur at a region that is particularly relevant for S100B-mediated RAGE signaling. Also, these observations suggest that the effects of the Nbs might also be exerted by selectively perturbing interactions between tetrameric S100B and specific domains in RAGE. Control experiments show that Nbs alone have no effect (Fig. 4*C*).

### Anti-S100B Nbs compete with the RAGE-VC1 domains for S100B binding

**Figure 5:**
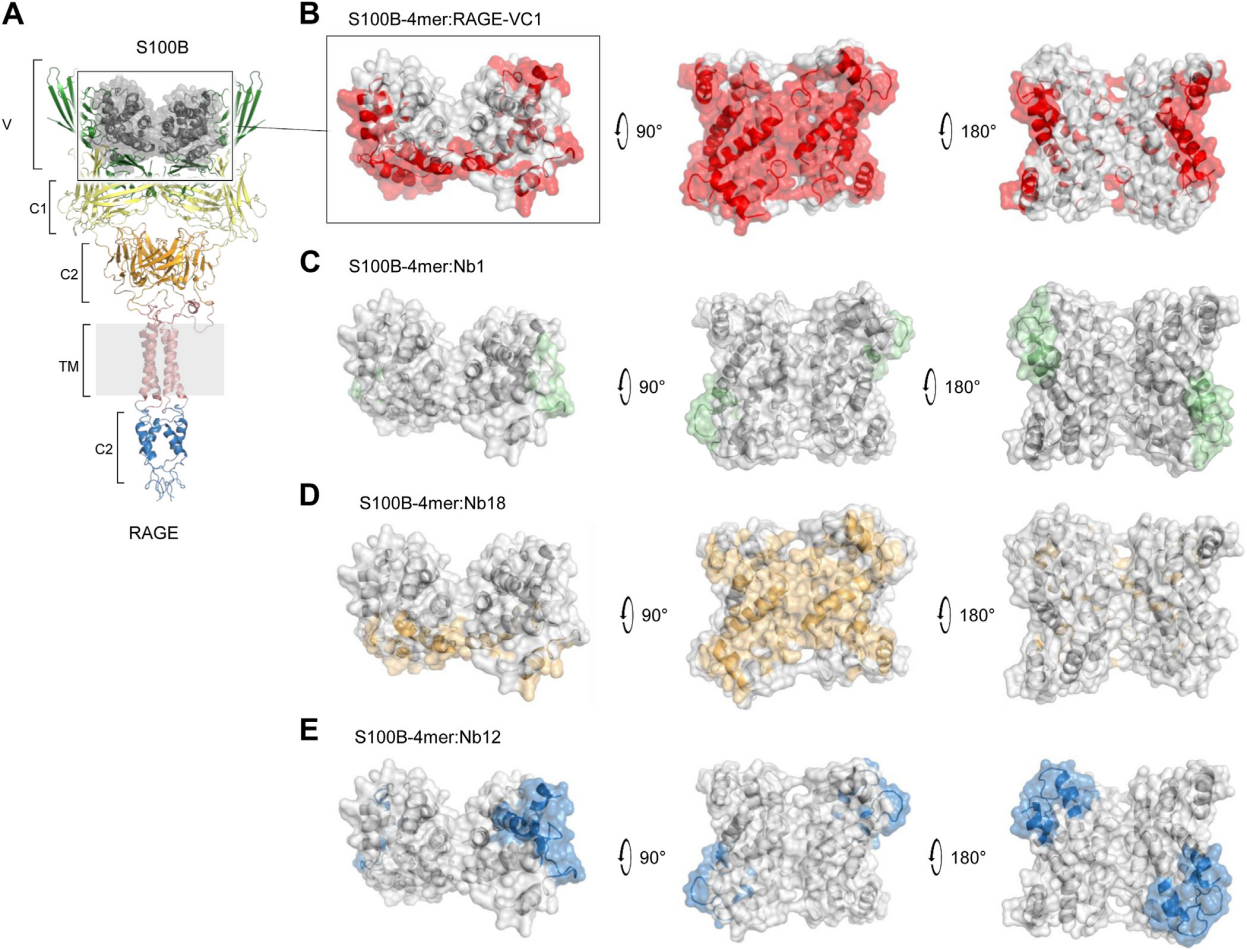
**Structural analysis of S100B–RAGE and S100B–nanobody (Nb) interactions.***A*, RAGE–S100B model (PDBDEV: 000080) depicting RAGE domains (*colored*) and RAGE bound tetrameric S100B (*gray*). *B*, mapping of RAGE V and C1 domain contacts on the surface of tetrameric S100B. *C–E*, mapping of Nb1, Nb18, and Nb12 contacts on the S100B tetramer structure to highlight overlapping regions of contacts with RAGE domains (see also Fig. S4). See Experimental procedures section for details. RAGE, receptor for advanced glycation end product.

To further dissect how Nbs influence RAGE engagement, we next explored how binding of the Nbs to S100B might inhibit the interaction of tetrameric S100B to specific domains in RAGE. For that, we resorted to a recent structural model of S100B-4mer–RAGE obtained from combining X-ray crystallographic data, mass spectrometry monitored cross-links, and molecular modeling (26) (Fig. 5*A*).

As evidenced in this model, and in agreement with previously described structures (7), the S100B–RAGE interaction involves the extended hydrophobic cleft of Ca^2+^ loaded S100B, formed by helices H_III_ and H_IV_, and the RAGE V and C1 domains (26) (Fig. 5*B*). To assess how Nbs might influence the S100B–RAGE interaction, we mapped contacts with tetrameric S100B (Fig. 5, *C*-*E*). This analysis revealed that the predicted contacts of Nb18 with S100B mostly overlap with regions in the central hydrophobic area of the tetrameric S100B that are involved in interactions with the RAGE-VC1 domains. In contrast, Nb1 interacts with the lateral sites of tetrameric S100B, which would disrupt contacts with the RAGE V-domain only. The same regions seem to be the preferred interaction sites for Nb12, albeit at very low affinity. Thus, we hypothesize that Nb1 and Nb12 specifically interfere with S100B–RAGE binding by blocking interactions with the RAGE V-domain, whereas Nb18 does so by blocking interactions with RAGE-VC1 domain.

**Figure 6:**
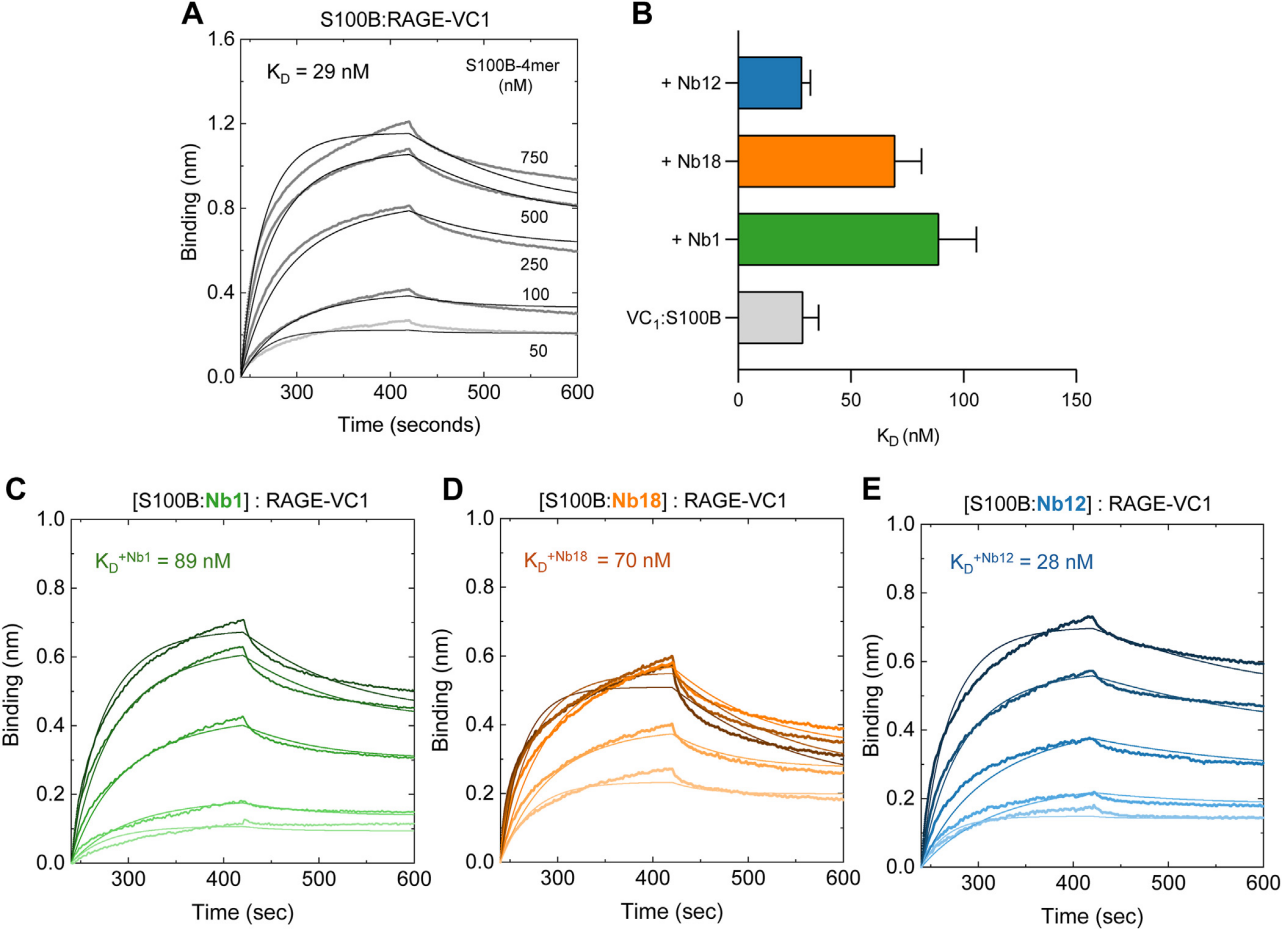
**Nanobodies (Nbs) binding to S100B-4mer compete for interactions with RAGE-VC1 domain.***A*, biolayer interferometry (BLI) sensograms of the interaction between S100B-4mer and biotinylated RAGE-VC1. *B*, effect of Nbs on the binding affinity of S100B-4mer to RAGE. BLI monitored competition assays for the binding between S100B-4mer and RAGE-VC1 in the presence of twofold excess of (*C*) Nb1, (*D*) Nb18, and (*E*) Nb12. RAGE, receptor for advanced glycation end product.

To experimentally test this possibility, we explored whether the anti-S100B Nbs could compete with RAGE-VC1 for binding to the S100B tetramer. For that, we performed BLI competition assays to monitor S100B binding to immobilized biotinylated RAGE-VC1, in the absence and presence of Nb1, Nb12, and Nb18 (Fig. 6).

RAGE-VC1 is an amenable model in interaction assays as it is the binding unit for almost all reported RAGE ligands, including S100B (27). Further, the RAGE V and C1 domains form a structural unit, which is more stable than the V-domain alone (6) making it a more suitable experimental model. In agreement with previous studies that established that RAGE-VC1 interacts at high affinity with Ca^2+^ bound tetrameric S100B (6, 7, 10), BLI analysis confirmed binding at nanomolar affinity (K_D_ ≈ 29 nM) (Fig. 6*A*). However, when tetrameric S100B was previously incubated with either Nb1 or Nb18, the equilibrium dissociation constant (K_D_) increased by 3-fold and 2.4-fold, respectively (Fig. 6, *B-D*). This denotes a decrease in binding affinity more significant with Nb1 than Nb18, which is consistent with the effects predicted from the structural analysis. On the other hand, Nb12 did not influence the binding affinity between S100B and RAGE-VC1, in agreement with its low binding affinity to S100B. Adequate control experiments revealed that none of the purified Nbs interacts with biotinylated RAGE-VC1.

## Conclusions

Our study successfully generated and characterized a library of Nbs against dimeric and tetrameric S100B, revealing that these Nbs can recognize epitopes common to both quaternary forms. Notably, we identified three Nbs—Nb1, Nb12, and Nb18—with distinct binding affinities and specificities. Through various biophysical analyses, we demonstrated that these Nbs bind S100B with high affinity, forming stable complexes, and can selectively modulate S100B's interaction with RAGE. This modulation was evidenced by the altered neurotrophic activity of S100B in SH-SY5Y cells and the competitive binding assays showing that Nbs, particularly Nb1 and Nb18, interfere with the S100B–RAGE interaction. These results match well with previous reports in which individual RAGE V and VC1 domains were used to sequester S100B before their interaction with cell surface RAGE, showing that the V-domain was able to reverse the increase in cell viability triggered by S100B, whereas the VC1 domain was slightly less efficient than the V-domain (10). Indeed, this is coherent with our data: Nb1 and Nb12 are more efficient in reverting cell viability increase triggered by S100B since they block mostly the binding of S100B to V-domain of RAGE, whereas Nb18 is less efficient because of a blockage of S100B to RAGE *via* the VC1 domain. Overall, these findings highlight the potential of anti-S100B Nbs as powerful investigational tools and therapeutic candidates. By elucidating the specific binding sites and modes of interaction between Nbs and S100B, we provide insights into the mechanisms by which these Nbs can modulate S100B functions. This opens up new avenues for the development of Nb-based therapeutics targeting S100B-related pathologies, particularly those involving neurodegenerative and inflammatory conditions.

## Experimental procedures

All reagents were of the highest grade commercially available. A chelex resin (Bio-Rad) was used to remove contaminant trace metals from all buffers.

### Expression and purification of human recombinant S100B protein

Human dimeric and tetrameric S100B were expressed in *E*. *coli* (BL21(DE3) E. Cloni EXPRESS; Lucigen) and purified to homogeneity as described (28, 29). The isolation and characterization of S100B oligomeric states (dimer and tetramer) were performed as described (8).

### Immunization and construction of an anti-S100B Nb library

Nbs targeting S100B were generated at the Nanobodies4Instruct platform in the VIB-VUB Center of Structural Biology in Brussels (21). Briefly, two *Llamas* were injected subcutaneously with 700 μg of recombinant S100B dimer or tetramer. Preimmune and immune sera were collected, and mRNAs were isolated from peripheral blood lymphocytes 4 days after the last injection. The mRNA was used as a template to create a complementary DNA library by reverse transcription. The VHH genes were amplified and ligated into a phagemid vector pMESy4 to express the Nbs fused with the phage coat proteins for display on phage. The display vector permits the inducible periplasmic expression of Nbs as soluble C-terminally His_6_-EPEA-tagged proteins in *E*. *coli strain* WK6.

### Selection of anti-S100B-specific Nbs

To screen for the presence of anti-S100B-specific Nbs, the library underwent three rounds of panning against immobilized S100B dimer and tetramer captured *via* their myc-tag. The antibody against the myc-tag was coated on the immunosorbent plates (Nunc; Maxisorp) overnight (ON) at 4 °C, to capture the S100B dimer–tetramer with a myc-tag. Phages were added to allow binding to S100B dimer or S100B tetramer. After the third round of panning, 96 individual clones from distinct selection outputs were selected and analyzed by ELISA for the presence of antigen-specific Nbs in periplasmic extracts.

### Expression and purification of anti-S100B Nbs

Anti-S100B Nbs were purified to homogeneity (21). pMESy4 vectors harboring Nb genes were transformed in *E*. *coli* WK6 cells, and the transformed cells were grown in Luria–Bertani medium supplemented with 2% glucose, 1 mM MgCl_2_, and 100 μg/ml of ampicillin at 37 °C, ON. The next day, 1% of the ON growth was added to Terrific Broth medium containing 0.1% glucose, 2 mM MgCl_2_, and 100 μg/ml of ampicillin and incubated at 37 °C, 200 rpm until absorbance is 0.7 to 1.2 at 600 nm. After an absorbance of 600 nm values were reached, expression was induced with 1 mM of IPTG ON at 25 °C, 200 rpm. The proteins in the periplasmic extract were recovered by osmotic shock and purified by immobilized metal affinity chromatography using an HisTrap HP column (Cytiva). The purified Nbs were buffer exchanged to 100 mM Tris–HCl, pH 7.4, and analyzed by SDS-PAGE.

### Dot-blot assays

About 2.5 μg of S100B dimer and tetramer were deposited in a 0.45 μm nitrocellulose membrane and left to dry ON. Next day, the membrane was blocked in 5% non–fat dry milk/Tris-buffered saline with Tween-20 (TBS-T) for 1 h followed by three cycles of 5 min wash with TBS-T. After the washing cycles, the membrane was incubated ON with 6 μM of the respective anti-S100B Nb to test. Next morning, three washing cycles of 5 min were performed followed by 2 h incubation with a secondary antipolyhistidine tag antibody conjugated with horseradish peroxidase (Sigma–Aldrich; A7058). After final washing, immunoreactivity was visualized using the Amersham ECL start Western Blotting Detection Reagent (Cytiva), and blots were imaged resorting to ChemiDoc XRS+ (Bio-Rad).

### Cross-reactivity assay

About 2.5 μg of several S100 proteins were deposited onto a 0.45 μm nitrocellulose membrane and left to dry ON. The S100 proteins tested were S100B dimer, S100B tetramer, S100A1, S100A2, S100A8, S100A9, S100A12, and S100P. BSA was used as a negative control. The next day, the membrane was blocked in 5% non–fat dry milk/TBS-T for 1 h followed by three cycles of 5 min wash with TBS-T. After the washing cycles, the membrane was incubated ON with 6 μM of Nb1, Nb12, and Nb18. Next morning, three washing cycles of 5 min were performed followed by 2 h incubation with a secondary antipolyhistidine tag antibody conjugated with horseradish peroxidase (Sigma–Aldrich; A7058). After final washing, immunoreactivity was visualized using the Amersham ECL start Western Blotting Detection Reagent (Cytiva), and blots imaged resorting to ChemiDoc XRS+ (Bio-Rad).

### CD

CD measurements were performed on a Jasco J-1500 spectropolarimeter equipped with a Peltier-controlled thermostated cell support at 25 °C. The anti-S100B Nb secondary structure was assessed by far-UV spectra (200–260 nm). The spectra were recorded at 0.1 mg/ml of protein concentration in 25 mM Tris–HCl, pH 7.4, using a 1 mm pathlength quartz cuvette (Hellma Analytics) and eight scans of average accumulation. The thermal stability was assessed by thermal unfolding with a linear temperature increase rate of one °C/min, from 20 to 98 °C, following ellipticity variation at 222 nm. The thermal curve normalization, fitting, and melting temperature estimations were performed in OriginPro version 2019 (OriginLab Corporation).

### FTIR

FTIR measurements were performed on a Bruker Tensor II FTIR Spectrometer (Billerica) equipped with a nitrogen-cooled MCT detector and a thermostated Harrick BioATR cell at 25 °C. Before spectra acquisition, 20 μl of each anti-S100B Nb (≈62 μM) in 5 mM sodium phosphate buffer were pipetted into the ATR cell and equilibrated for 5 min. FTIR spectra between 900 and 4000 cm^−1^ were acquired with 120 technical accumulations, 12 mm of aperture, 20 kHz scanner velocity, 4 cm^−1^ spectral resolution, and buffer background correction.

### Tryptophan fluorescence spectroscopy

Fluorescence measurements were performed on a Jasco FP-8200 spectrofluorometer (Jasco, Inc) equipped with a Peltier-controlled thermostatic cell support. Tryptophan emission spectra were recorded from 300 nm to 450 nm, using 10 nm of emission slits and 5 nm of excitation slits upon 280 nm excitation in low sensitivity. The tryptophan’s emission spectra were recorded with 1 μM of anti-S100B Nbs in 25 mM Tris–HCl, pH 7.4. The thermal stability was assessed by changes in the tryptophan’s microenvironment with a linear temperature increase rate of 1 °C/min, from 20 to 95 °C, following emission at 330 nm and 350 nm. The thermal curve normalization, fitting, and melting temperature estimations were performed in Origin 2019.

### Biolayer interferometry

BLI assays were performed on an Octet N1 (Sartorius) at 25 °C with a shaking speed of 2200 rpm. Protein samples for analysis were prepared in kinetic buffer (50 mM Tris–HCl [pH 7.4] and 150 mM NaCl). The sensograms were obtained with a 30 s baseline, 180 s loading, 30 s baseline, 180 s association, and 240 s dissociation. Anti-S100B Nbs were diluted in kinetic buffer to a final concentration of 20 μg/ml and loaded onto a Ni–NTA biosensor (Sartorius). S100B dimer and tetramer was diluted in kinetic buffer to a final concentration between 250 nM and 25 nM. The sensograms were corrected by performing a run with kinetic buffer. Raw data were analyzed in Octet N1 (Sartorius) data analysis software. The curves were corrected at the start of association and dissociation; a global fitting (1:1) was performed, and the values of $k_{a}$, $k_{d,}$ and $K_{D}$ were calculated.

### Analytical SEC

Analytical SEC of Nb-S100B dimer and Nb-S100B tetramer were performed at room temperature on a Superdex 75 Tricorn high performance column (Cytiva, bed volume = 26 ml) and Superdex 200 Tricorn (bed volume = 25.2 ml), respectively. The protein was eluted at 1 ml/min with 50 mM Tris–HCl (pH 7.4), 150 mM NaCl (+CaCl_2_). 50 μM of S100B dimer, or 100 μM of S100B tetramer (dimer equivalent) was incubated with 50 μM of anti-S100B Nb in the presence and absence of 1.1 mM CaCl_2_ for 30 min at room temperature and injected. Controls were performed for the Nb and S100B dimer–tetramer alone in the same conditions. The fractions corresponding to the complex were analyzed by SDS-PAGE.

### Cell culture and cell viability assays

The human neuroblastoma cell line SH-SY5Y (American Type Culture Collection; CRL-2266) was maintained in DMEM-F12 medium supplemented with 10% fetal bovine serum, 1% streptomycin–penicillin, and 0.1% amphotericin B in a humidified incubator with 5% of carbon dioxide. For cell viability assays, SH-SY5Y cells were plated in a 96-well culture plate (10,000 cells/well) and grown for 24 h in DMEM-F12 medium supplemented with 10% fetal bovine serum and 1% streptomycin–penicillin. After 24 h, cells were serum-starved for an additional 24 h before treatment. The next day, cells were treated with solvent vehicle control (buffer; 50 mM Hepes [pH 7.4]), 1% Triton X-100, and a combination of 5 μM of S100B tetramer (dimer equivalent) with 5 μM or 10 μM of Nb1, Nb12, and Nb18 for 48 h. Controls were also performed with 10 μM of Nb1, Nb12, and Nb18. After 48 h, the cells were incubated with 1x of resazurin dye for 3 h. Fluorescence of converted resorufin was measured, and the normalized values to the vehicle control were represented as cell viability. All data are expressed as cell viability (normalized to buffer) with the individual points of at least three independent experiments. Statistical analysis was performed by normalization test followed by one-way ANOVA.

### Docking and intermolecular contact analysis

Protein structures were visualized and manipulated in PyMOL (version 2.4.1; Schrödinger). Ca^2+^-S100B octamer crystallographic structure was obtained in Protein Data Bank (PDB ID: 2H61) and stripped of water molecules, whereas the S100B tetramer was obtained from the structure by deleting dimers EF and GH (7). The Ca^2+^-S100B dimer crystallographic structure was obtained in Protein Data Bank (PDB ID: 3D0Y) in Set/2023 (30). The model of RAGE-S100B hetero-octamer was obtained in PDB-Dev (PDBDEV: 00000080) (26). The anti-S100B Nb1, Nb12, and Nb18 were obtained by inputting the amino acid sequence in NanoBodyBuilder 2 (University of Oxford) (24). Docking calculations of the Nbs and S100B dimer–tetramer were done using the antibody mode of ClusPro 2.0 web server (Boston University) (31, 32, 33, 34, 35). For the anti-S100B Nbs, a masking file containing non-CDRs was provided. The best docking model (highest cluster number) was considered for analysis. The intermolecular contacts of RAGE–S100B-4mer, Nb1–S100B-4mer, Nb18–S100B-4mer, and Nb12–S100B-4mer complexes were mapped resorting to MAPIYA (Contact Map Server) (36) by inputting the PDB structures of the respective complexes.

### Expression and purification of VC1 RAGE

The VC1 domain of human RAGE was expressed in Rosetta-gami B (DE3) (Novagen) and purified to homogeneity (6).

### Competition assays by BLI

BLI assays were performed on an Octet N1 (Sartorius) at 25 °C with a shaking speed of 2200 rpm. Protein samples for analysis were prepared in kinetic buffer (PBS, 0.1% BSA, 0.05% Tween-20, and 1.1 mM CaCl_2_). The sensograms were obtained with a 30 s baseline, 180 s loading, 30 s baseline, 180 s association, and 240 s dissociation. RAGE-VC1 was biotinylated resorting to EZ-Link Sulfo-NHS-Biotin (A39256; Thermo Fisher). Biotinylated RAGE-VC1 was diluted in kinetic buffer to a final concentration of 25 μg/ml and loaded into a streptavidin biosensor (Sartorius). RAGE-VC1 was then dipped in different concentrations of S100B tetramer (ranging from 750 nM to 50 nM) in the absence or presence of previously incubated at twofold excess of each anti-S100B Nbs. The sensograms were corrected by performing a run with kinetic buffer. Raw data were analyzed in Octet N1 (Sartorius) data analysis software. The curves were correct at the start of association and dissociation, and a global fitting (1:1) was performed and the values of $k_{a}$, $k_{d,}$ and $K_{D}$ were calculated.

## Data availability

All data generated and analyzed in this study are included in this article and its supporting information file.

## Supporting information

This article contains supporting information.

## Conflict of interest

The authors declare that they have no conflicts of interest with the contents of this article.
