# Supplementary material for: Functional modulation of RAGE activation by multimeric S100B using single-domain antibodies
Source: J Biol Chem. 2024 Nov 13;300(12):107983. doi: 10.1016/j.jbc.2024.107983 (PMC11667159; doi:10.1016/j.jbc.2024.107983)
Supplement: Supplemental Fig. S1-S5 [file mmc1.docx]

# Supporting Information

**Functional modulation of RAGE activation by multimeric S100B using single-domain antibodies**

Margarida C. Simões^1,2^, Joana S. Cristóvão^1,2^, Els Pardon^3,4^, Jan Steyaert^3,4^, Günter Fritz^5^, Cláudio M. Gomes^1,2*^

^1^ BioISI – Instituto de Biosistemas e Ciências Integrativas, Faculdade de Ciências, Universidade de Lisboa, 1749-016 Lisboa, Portugal

^2^ Departamento de Química e Bioquímica, Faculdade de Ciências, Universidade de Lisboa, 1749-016 Lisboa, Portugal

^3^ Structural Biology Brussels, Vrije Universiteit Brussel (VUB), Pleinlaan 2, B-1050 Brussels, Belgium

^4^ VIB-VUB Center for Structural Biology, VIB, Pleinlaan 2, B-1050 Brussels, Belgium.

^5^ Department of Cellular Microbiology, University of Hohenheim, Stuttgart 70599, Germany

Supporting Material Contents:

1. Figure S1. **CD-monitored nanobody thermal stability** (Page S-2).
2. Figure S2. **Binding and structural analysis of three anti-S100B Nbs to S100B-2mer** (Page S-3).
3. Figure S3. **Calcium does not influence Nb binding to S100B** (Page S-4).
4. Figure S4. **Mapping computed Nb:S100B interactions** (Page S-5).
5. Figure S5. **Anti-S100B Nbs compete with RAGE-VC1 for S100B-4mer binding** (Page S-6).


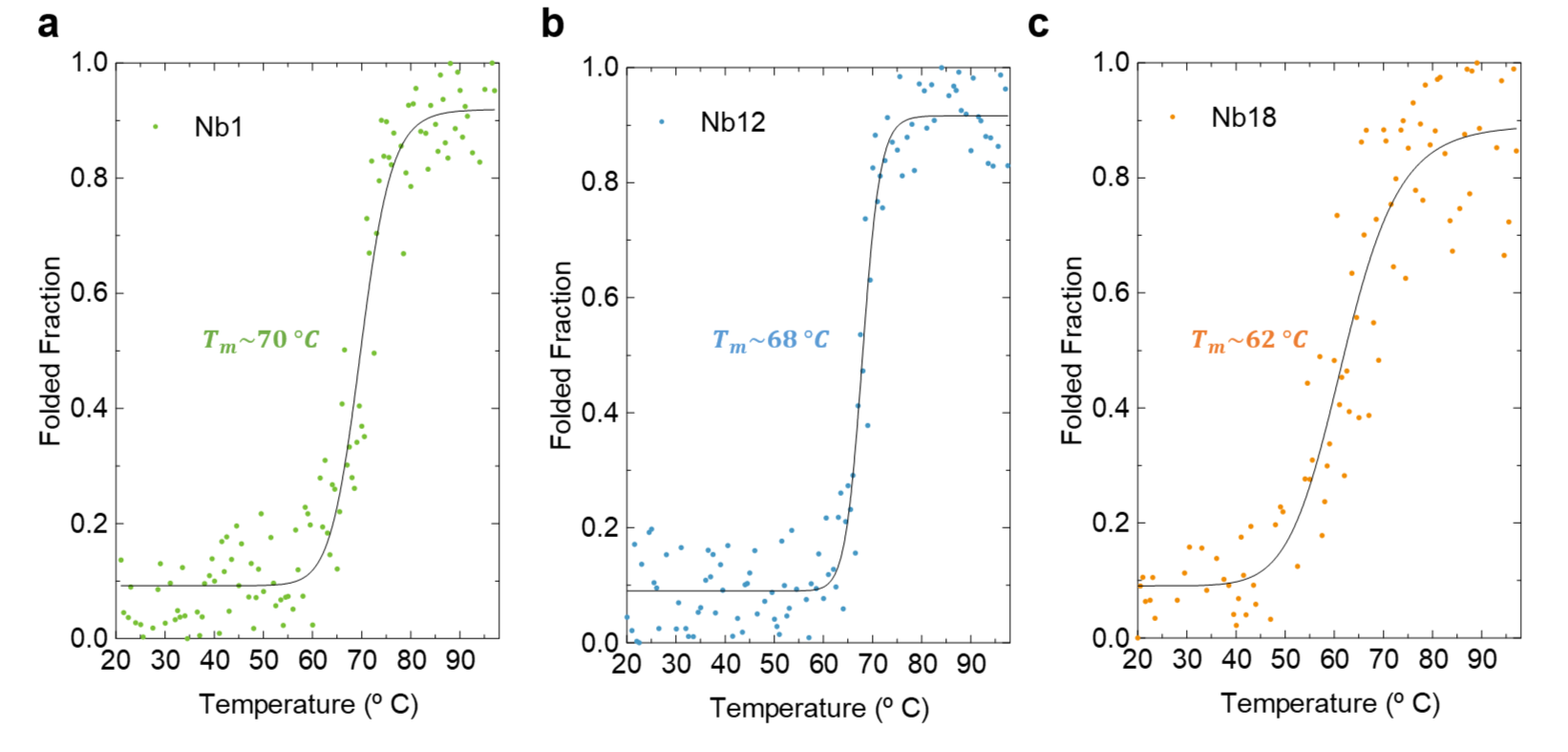


**Figure S1 – CD-monitored nanobody thermal stability.** Thermal denaturation curves monitored by CD spectroscopy at 222 nm and estimated melting temperature (T_m_) of a) Nb1, b) Nb12 and c) Nb18.

**
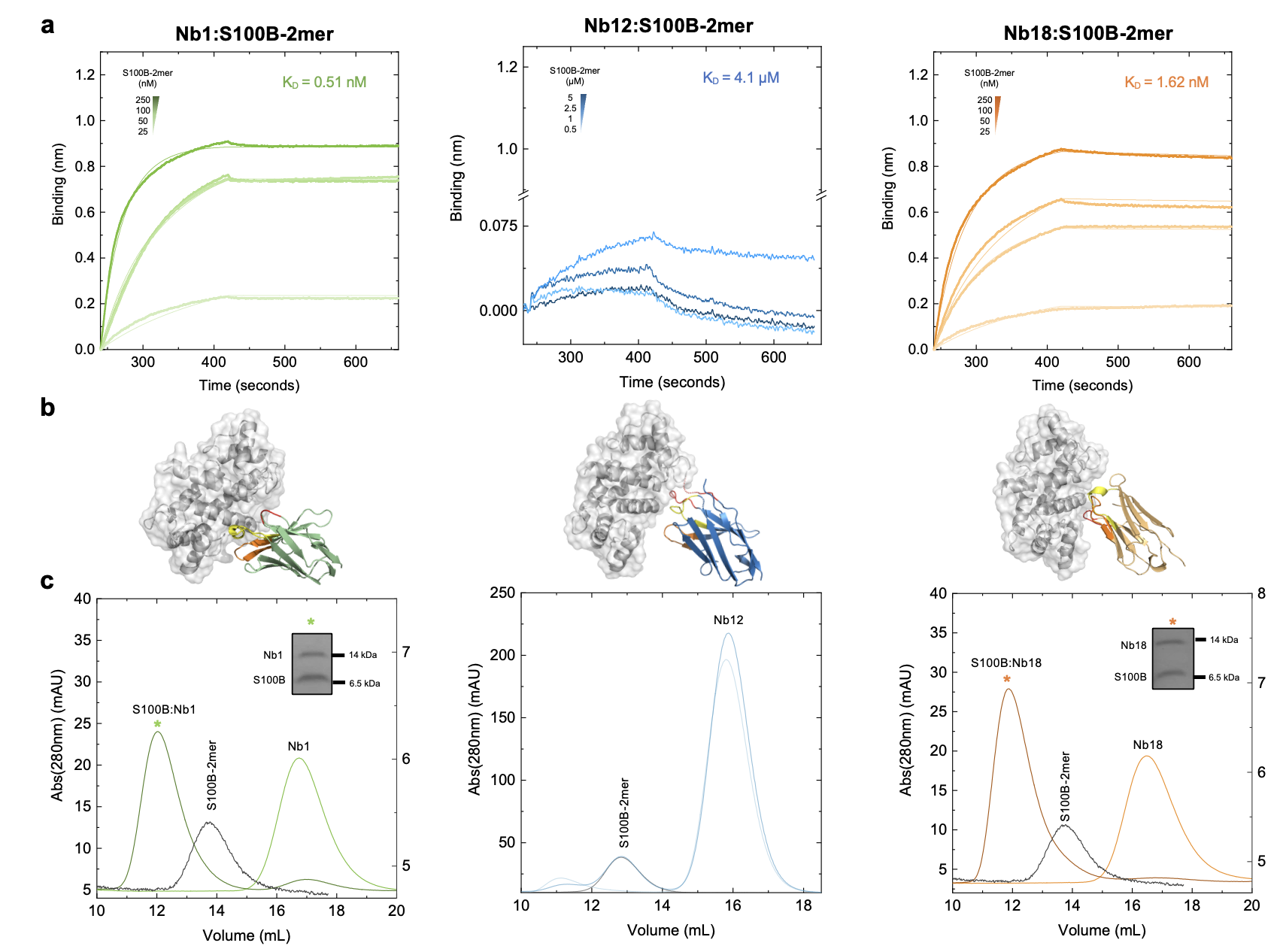
**

**Figure S2** – **Binding and structural analysis of three anti-S100B Nbs to S100B-2mer.** **a)** BLI sensorgrams and fitting of Nb1 (green), Nb12 (blue) and Nb18 (orange) binding to S100B-2mer. **b)** Docking models of complexes between Nbs (coloured, Nb1 in green, Nb12 in blue and Nb18 in orange) and S100B-2mer (grey). **c)** Chromatograms obtained from size-exclusion chromatography resolved Nbs, S100B-2mer and binary complexes (for Nb1 and Nb18); inset depicts lane from an SDS-PAGE obtained from the peak corresponding to the complex (*), to identify the presence of Nbs and S100B. No Nb12:S100B-2mer complex was observed. See materials and methods for details.


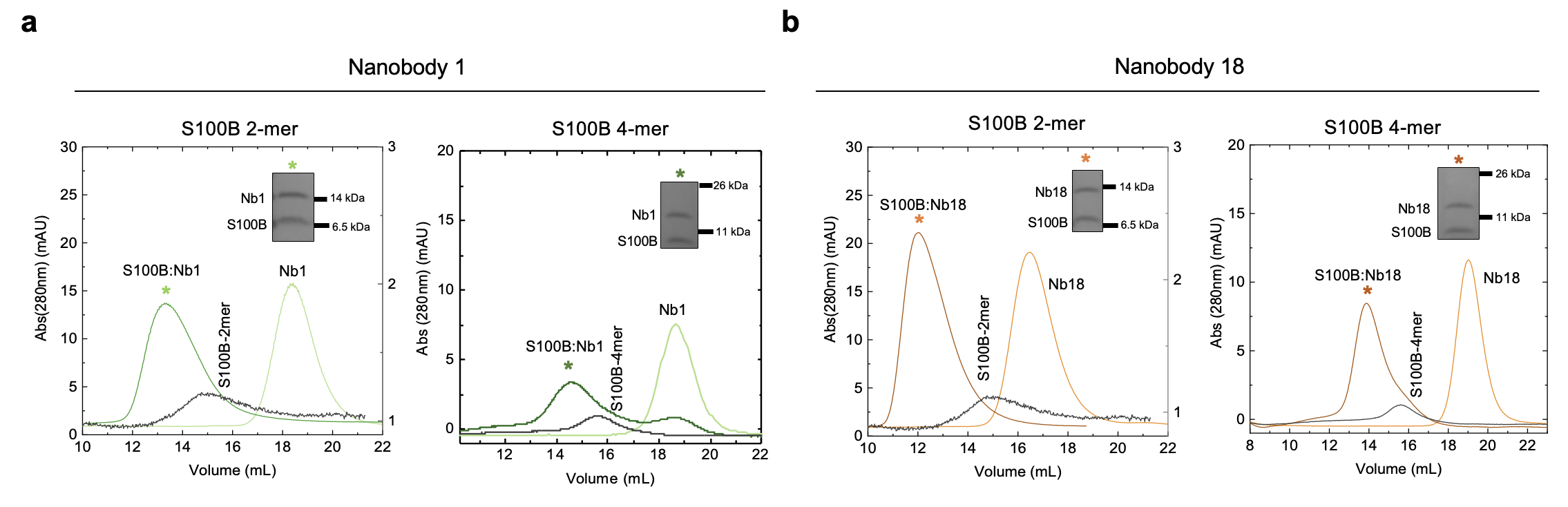


**Figure S3: Calcium does not influence Nb binding to S100B.** a) Chromatograms obtained from size-exclusion chromatography of Nb:S100B-2mer and Nb:S100B-4mer complexes in the presence of Ca^2+^; inset depicts lane from an SDS-PAGE obtained from the peak corresponding to the complex (*), to identify the presence of Nb1 and S100B. b) Chromatograms obtained from size-exclusion chromatography resolved Nb18, S100B-2mer and S100B-4mer; inset depicts lane from an SDS-PAGE obtained from the peak corresponding to the complex (*), to identify the presence of Nb18 and S100B.

**
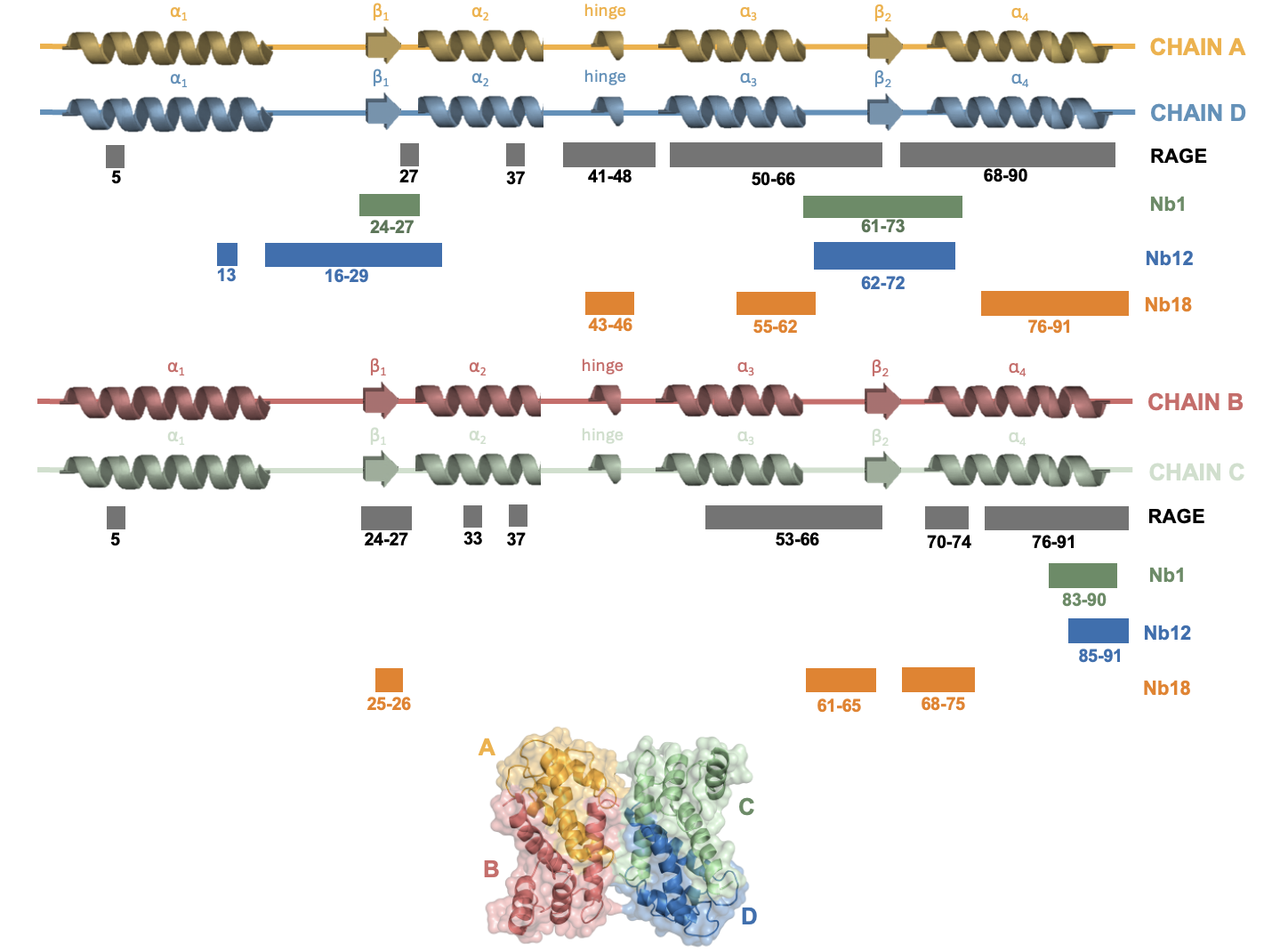
**

**Figure S4: Mapping computed Nb:S100B interactions.** Nb:S100B contacts obtained from docking solutions are represented by boxes below the S100B sequence. S100B chains are colour coded and labelled A-D as in the represented structure (adapted from PBD ID 2H61).


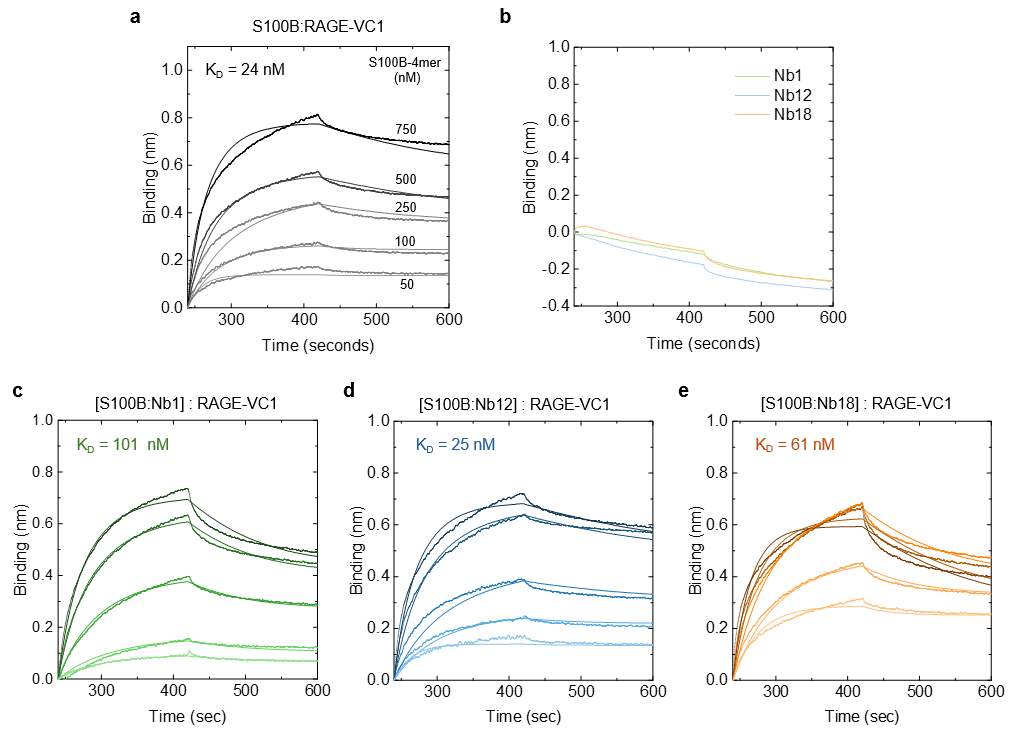


**Figure S5: Anti-S100B Nbs compete with RAGE-VC1 for S100B-4mer binding.** Biolayer interferometry assay for the interaction of a) S100B-4mer and RAGE-VC1 domain (grey) (replicate). b) RAGE-VC1 domain and Nb1 (green), Nb12 (blue) and Nb18 (orange). c) S100B-4mer and RAGE-VC1 domain in the presence of Nb1 (green) (replicate). d) S100B-4mer and RAGE-VC1 domain in the presence of Nb12 (blue) (replicate). e) S100B-4mer and RAGE-VC1 domain in the presence of Nb18 (orange) (replicate).
